# Strategies to Prevent Preterm Birth

**DOI:** 10.3389/fimmu.2014.00584

**Published:** 2014-11-19

**Authors:** John P. Newnham, Jan E. Dickinson, Roger J. Hart, Craig E. Pennell, Catherine A. Arrese, Jeffrey A. Keelan

**Affiliations:** ^1^School of Women’s and Infants’ Health, The University of Western Australia, Perth, WA, Australia

**Keywords:** preterm birth, prevention, progesterone, smoking, pregnancy

## Abstract

After several decades of research, we now have evidence that at least six interventions are suitable for immediate use in contemporary clinical practice within high-resource settings and can be expected to safely reduce the rate of preterm birth. These interventions involve strategies to prevent non-medically indicated late preterm birth; use of maternal progesterone supplementation; surgical closure of the cervix with cerclage; prevention of exposure of pregnant women to cigarette smoke; judicious use of fertility treatments; and dedicated preterm birth prevention clinics. Quantification of the extent of success is difficult to predict and will be dependent on other clinical, cultural, societal, and economic factors operating in each environment. Further success can be anticipated in the coming years as other research discoveries are translated into clinical practice, including new approaches to treating intra-uterine infection, improvements in maternal nutrition, and lifestyle modifications to ameliorate maternal stress. The widespread use of human papillomavirus vaccination in girls and young women will decrease the need for surgical interventions on the cervix and can be expected to further reduce the risk of early birth. Together, this array of clinical interventions, each based on a substantial body of evidence, is likely to reduce rates of preterm birth and prevent death and disability in large numbers of children. The process begins with an acceptance that early birth is not an inevitable and natural feature of human reproduction. Preventative strategies are now available and need to be applied. The best outcomes may come from developing integrated strategies designed specifically for each health-care environment.

## Introduction

Each year, 15 million babies are born preterm (1). Many may look forward to a normal life, but others may die or live a life of disability. Worldwide the rate of preterm birth is 11.1% but varies with geography and race, ranging from 15% or more in some parts of Africa to 5–6% in several European nations (2) and possibly lower in some parts of East Asia (2).

In most countries, the rate of preterm birth has risen in recent decades and worldwide now represents the largest cause of neonatal death (3) and the second largest direct cause of death in children up to 5 years of age (2). Discovering how to lower the rate of this major complication of pregnancy needs to be one of the highest priorities in contemporary health care.

Prevention of early birth, however, presents several great challenges. The condition merely describes an event that occurs before its due time, and is not a diagnosis in itself. There are many pathways leading to preterm birth and the prevention of each requires different types of scientific inquiry and clinical strategies, which together encompass a wide array of measurement systems and clinical interventions across many health-care disciplines (4).

With such a great challenge, what evidence do we have that prevention of preterm birth is possible and feasible?

## Lessons from Populations in Transition

Changing rates of preterm birth in populations in transition provide some evidence that environmental and lifestyle factors may be involved, and these may be amenable to intervention.

In China, the rate of preterm birth is not known with certainty as there is no national obstetric data reporting system, but the estimated rate is thought to be relatively low by international standards. Hospital-based reports suggest rates ranging from 3 to 6% (5–8). A geographic-based study employing ultrasound confirmation of gestational age in early pregnancy in Jiangsu Province indicated rates of 2.6 and 2.9% in urban and rural regions, respectively (9). These rates appeared to rise as Chinese women lived in increasingly Westernized environments, with preterm birth rates of 4.4% in China-born women in Western Australia; 5.6% in non-resident Chinese women living in Hong Kong; and 7.6% for Hong Kong women with residency status. The factors underpinning these differing rates suggest that environment and lifestyle may be involved in modifying preterm birth rates and that factors operating outside traditional China may somehow have increased the rate by several percentage points. These figures provide an indirect clue as to the potential magnitude of the effect of preventative strategies, at least in women of Chinese origin living in Western environments.

Mexican women after migration to USA also experience an increase in risk of preterm birth. Long-term immigrants who had lived in USA for more than 5 years were shown to have a 1.9-fold greater risk of delivering preterm, and a 1.5 greater risk of giving birth to a low-birth weight infant when compared with more recent arrivals (10). The factors involved in the increasing preterm birth risks in these population groups are uncertain, but long term Mexican immigrant women have been shown to have higher parity, more pregnancy complications, fewer planned pregnancies, and to smoke. An almost doubling in preterm birth rate as these lifestyle factors are adopted by immigrant women suggests that appropriate interventions may reduce the rate of early birth by nearly half.

## Opportunities to Prevent Preterm Births

“Green shoots” are now appearing in the field of preterm birth prevention. At least in high-resource settings, some strategies are suitable for immediate use in clinical practice, have a high likelihood of success, and in many regions have already been adopted in part or in whole. Other strategies have potential and feasibility, but evidence for their effectiveness is still uncertain. Finally, there are initiatives in place aimed at other clinical end-points but which may incidentally prevent some cases of early birth.

The levels of evidence employed in this review and that enable description of potential effectiveness in high-resource settings are shown in Table 1, and the potential strategies that are feasible and suitable for implementation are shown with their level of evidence in Table 2.

**Table 1:** **Levels of evidence for intervention studies as used by the Australian National Health and Medical Research Council (NHMRC) and employed in this review**.

Level	Intervention
I	Systematic review of level II studies
II	Randomized controlled trial
III-1	Pseudo-randomized controlled trial (i.e., alternate allocation or some other method)
III-2	Comparative study with concurrent controls
	Non-randomized experimental trial
	Cohort study
	Case-control study
	Interrupted time series with control group
III-3	Comparative study without concurrent controls
	Historical control study
	Two or more single-arm study
	Interrupted time series without a parallel control group
IV	Case series with either post-test or pre-test/post-test outcomes

https://www.nhmrc.gov.au/_files_nhmrc/file/guidelines/developers/nhmrc_levels_grades_evidence_120423.pdf

**Table 2:** **Strategies to prevent preterm birth feasible for implementation and likely to be successful in high-resource settings**.

Strategy	Possible reduction in PTB	Level of evidence
Prevent non-medically indicated late preterm/early term birth	55% (11)	III-3
Progesterone supplementation	45% (12)	I
Cervical cerclage	20% (13)	III-1
Tobacco control
Prevent smoking in pregnancy	20% (14)	III-2
Smoke-free legislation	10% (15)	III-3
Judicious use of fertility treatments	63% (16)	I
Dedicated preterm birth prevention clinics	13% (17)	III-2

*Levels of evidence as defined in Table 1*.

## Strategies Suitable for Immediate Use

There are six strategies currently available with various levels of evidence of effectiveness that are suitable for translation into clinical practice in high-resource settings and have a high chance of successfully preventing a proportion of preterm births.

### Preventing non-medically indicated late preterm birth

The most feasible approach to rapidly lowering the overall rate of preterm birth is to address non-medically indicated late preterm birth. Late preterm birth is defined as birth between 34 weeks 0 days and 36 weeks 6 days and these infants account for 70% of all preterm births (18). In USA, the rate of preterm birth increased by one-third over the last 25 years and this increase has resulted almost entirely from a rise in late preterm births (19). In the period 1990–2006, the late preterm birth rate for singleton births increased 20% from 6.7 to 8.1%. Similar increases in late preterm birth have been described in other countries during this time period, including South America (20), France (21), and Australia (22, 23).

Late preterm birth is a potential danger to the child. Infants born in the late preterm period are physiologically and metabolically immature. Their brain mass is approximately 70% of that of a term infant and the ongoing process of myelination is reduced accordingly (24). In the neonatal period, late preterm infants are at increased risks of death, admission to neonatal intensive care, respiratory distress and need for mechanical ventilation (25), apnea, temperature instability, hypoglycemia, hyperbilirubinemia, poor feeding, separation from their mother, and re-admission after discharge (24). In childhood, late preterm infants are at increased risk of death (18), cerebral palsy (26), speech disorders (27), growth delay and stunting (28), developmental delay (29), behavioral problems including attention deficit disorder (24, 30, 31) and learning difficulties (31, 32). The financial costs are considerable, not just for the health-care system in the short term, but for the individual, the family, and the society in terms of life-long productivity.

Recently, the effect of birth in the early term period has received increasing attention (33). Early term birth has been defined as birth between 37 weeks 0 days and 38 weeks 6 days gestation (24) and accounts for approximately 17% of all births. Rates of early term birth have risen in recent years in many regions. Neonatal and infant morbidities are increased in the early term period, when compared with births at 39 weeks gestation or more, but the magnitude of excess risk is less than in the late preterm birth age group. As an example, a recent Canadian study observed that the adjusted relative risk for admission to neonatal intensive care was 6.14 (95% CI 5.63, 7.03) for late preterm birth and 1.54 (95% CI 1.41, 1.68) for early term birth (34). For neonatal respiratory morbidity, the adjusted relative risks were 6.16 (95% CI 5.39, 7.03) and 1.46 (95% CI 1.29, 1.65), respectively. In a comparison of outcomes after planned Cesarean section in 19 centers in USA, it was observed that when compared with births at 39 completed weeks, rates of admission to a neonatal intensive-care unit (NICU), need for mechanical ventilation, and treatment for sepsis or hypoglycemia were increased 1.8- to 4.2-fold for births at 37 weeks and 1.3–2.1 for births at 38 weeks (35).

A significant proportion of all late preterm and early term births result from obstetric or medical complications of pregnancy, while others may be precipitated out of concern for maternal or fetal well being. As a result, assessment of outcomes after birth is confounded by the relative contributions of prematurity and any abnormalities in the pregnancy that contributed to the early gestational age at birth. The role of such biological determinants has been investigated and found to be amplified at earlier gestational ages, but quantification of the true relative contributions of gestational age and biological determinants remain challenging and will require further investigation (34).

Strategies addressing the increase in late preterm and early term births will be enhanced by an understanding of the demographic characteristics of those most at-risk. In a review of the factors contributing to the rise in preterm birth rates at 36 and 37 weeks gestation in USA between 1992 and 2002, non-Hispanic white births were found to be the greatest contributor (36). Rates of early preterm birth in Hispanic and black births remained relatively constant. The factors underpinning the rise in non-Hispanic white early births is likely to be socioeconomic and include access to health care and possibly maternal request for intervention.

If then the major contributor to rising preterm birth rates in recent years has been late preterm birth, is it possible to intervene and can it be done with safety? The clinical reasoning behind many cases of early intervention is to prevent stillbirth. In a 10-year population-based study in New South Wales, Australia encompassing more than 100 hospitals and approximately one-third of all births in Australia, the number of planned interventions before the estimated due date was found to have increased to 26% of all singleton births >32 weeks gestation (22). Stillbirth rates were unchanged over this time period, indicating that at least at a population level, the increased intervention did not improve infant survival.

Researchers in Denmark have reported the outcomes of the first randomized controlled trial of elective Cesarean section planned for 38 weeks, versus 39 weeks, gestation (37). An original sample size of 1010 participants was increased to 1270 when a high rate of non-compliance was observed. There were small reductions in rates of admission to NICU in the delayed delivery group, but the differences were not statistically significant. Interpretation of these findings is confounded by uncertainty as to whether the study was under-powered and by the unexpected high rate of earlier delivery in the planned later birth group (23).

The effects of introducing policies to lower the rate of elective delivery before 39 weeks gestation have been reported as a retrospective cohort of prospectively collected data (11). The study involved 27 facilities across 14 states in USA. After an education campaign and declaration of an intent to reduce non-medically indicated birth <39 weeks gestation on the basis of patient safety, medical staff were allowed to choose one of three approaches: (1) a “hard stop” approach in which elective delivery <39 weeks would be refused by hospital staff and the policy would be enforced; (2) a “soft stop” approach in which compliance would be left to individual clinicians but that any departure from policy would be referred to a local peer review committee for “evaluation and potential action”; and (3) an “education only” approach.

During the study period, the rate of elective delivery between 37 and 39 weeks gestation fell significantly from 9.5% of all births in 2007 to 4.3% of births in 2009. The rate of elective early term delivery was significantly reduced in both the “hard stop” and “soft stop” groups, and the reduction in the “hard stop” group was double that in the “soft stop” group. There was a small decline in the “education only” group, but the difference did not achieve statistical significance. Interestingly, there was considerable variation between facilities in their rates of elective delivery <39 weeks before the intervention commenced. Each facility had self-selected their choice of the three strategies. The large reduction in rates of early birth in some facilities, which began with high levels of intervention indicates that any such intervention may be most effectively targeted at those with the highest rate of early intervention. Overall, in the study facilities during the observation period, the rate of term newborn intensive care admission fell 15% from 8.9 to 7.5% (CI 0.79, 0.92), while stillbirths rates were unchanged.

The findings of this intervention cohort study provide strong evidence that the rate of late preterm and early term birth may be lowered and result in a 15% reduction in admissions to neonatal intensive care. Such an approach does not appear to increase the rate of stillbirth. Education alone did not significantly improve outcomes, but the process of education was aimed solely at the health-care providers, and it is yet to be seen if a combined approach of providing education to both the women themselves and the health-care personnel may result in a more favorable outcome.

The price for obstetricians and their hospitals of delaying birth, however, is an increase in the number of births out-of-hours. In a secondary analysis of the Danish randomized controlled trial of planned Cesarean birth at 38 or 39 weeks gestation (38), planned delivery at 39 weeks compared with 38 weeks resulted in a 60% increase in unscheduled Cesarean sections and a 70% increase in deliveries outside regular working hours. Further research is urgently required to determine the most effective strategies by which late preterm and early term births may be minimized without deleterious impacts on the health-care system and while maintaining patient safety. Future strategies may be most effective if they recruit to the cause not just the health-care professionals but also the pregnant women and their families.

### Progesterone supplementation

For several decades, there has been interest in the potential use of progesterone supplementation to prevent preterm birth but a series of recent studies has now provided strong evidence for their usefulness.

Ironically, the mechanism by which progesterone may delay birth remains uncertain. In non-primate placental mammals, the uterine quiescence of pregnancy is maintained by high circulating levels of progesterone and falling levels herald the onset of labor (39). In human beings, there is no such decline in circulating levels before labor (40). Two possible mechanisms of action are proposed. First, progesterone has an anti-inflammatory action that may counteract the inflammatory process that is involved in initiation of labor (41). Second is a possible functional withdrawal of progesterone through changes in progesterone receptors and their transcriptional activity at a tissue level (41–43).

Progesterone has been administered in several formulations. For preterm birth prevention, natural progesterone is used (44). Synthetic progesterones, such as medroxyprogesterone acetate are not used as they have significant androgenic activity. Natural progesterones can be given vaginally, orally, or by injection. Vaginal progesterone has the advantage of being locally available and has few side effects although some women complain of vaginal irritation. The half-life is 13 h (45) and daily treatment is required. Various doses are employed ranging from 90 to 400 mg but there is no evidence that any one dose is superior to another. An alternative agent is 17 α-hydroxy-progesterone (17P) caproate, which is also a natural progesterone conjugate but with a longer half-life of 7 days (44). 17P is administered intramuscularly and is given once each week. Both these progesterone formulations are considered to be safe in pregnancy.

In women with a past history of preterm birth, progesterone has been shown in meta-analysis of RCTs to significantly reduce the risk of preterm birth <34 weeks (RR 0.31 95% CI 0.14–0.69), preterm birth <37 weeks (RR 0.55, 95% CI 0.42–0.74), perinatal death (RR 0.50, 95% CI 0.33–0.75), need for assisted ventilation (RR 0.40, 95% CI 0.18–0.90), necrotizing enterocolitis (RR 0.30, 95% CI 0.10–0.89), and admission to neonatal intensive care (RR 0.24, 95% CI 0.14–0.40) (46).When given to women with a past history of preterm birth, there is no evidence for a difference in effectiveness between daily natural vaginal progesterone and weekly intramuscular 17P injections. As a result of these findings, the American College of Obstetricians and Gynecologists recommends that “progesterone supplementation for the prevention of recurrent preterm birth should be offered to women with a singleton pregnancy and a prior spontaneous preterm birth due to spontaneous labor or premature rupture of membranes” (47).

There is also strong evidence that progesterone treatment may prevent preterm birth in women shown to have a short cervix on ultrasound imaging in mid-pregnancy. Meta-analysis of individual patient data of five trials has shown that vaginal progesterone given to pregnant women in the mid-trimester with a short cervix ( ≤25 mm) is associated with a significant reduction in the rate of preterm birth <28 weeks (RR 0.50, 95% CI 0.30–0.81), <33 weeks (RR 0.58, 95% CI 0.42–0.80) and <35 weeks (RR 0.69, 95% CI 0.55–0.88), in addition to significant reductions in risk of newborn complications including respiratory distress syndrome, need for mechanical ventilation, admission to neonatal intensive care, and composite morbidity and mortality (48).

If progesterone is so effective in preventing preterm birth in women with a short cervix in mid-pregnancy, should all pregnant women be screened for cervical length at this time? The question is of great importance and remains controversial. In one of the major and most important trials in this field, Hassan and colleagues allocated at random asymptomatic women with a singleton pregnancy and short cervix (10–20 mm) between 19 weeks 0 days and 23 weeks 6 days to receive either vaginal progesterone gel or placebo daily (12). Preterm birth and the major complications of prematurity were halved by the treatment, consistent with the findings from other studies. The numbers of women, however, required to achieve this reduction were large. A total of 32,091 pregnant women were screened to identify 733 with a cervix length between 10 and 20 mm. 268 women declined to participate or were excluded, leaving 236 randomized to the treatment group and 229 to the placebo. The primary outcome of birth <33 weeks was observed in 21 cases in the treatment group (8.9%) and 36 cases in the placebo group (16.1%), preventing the early birth of 15 cases out of 36 eligible. Therefore, if we were to assume that introduction into clinical practice were to involve administration of vaginal progesterone to all women with a cervix between 10 and 20 mm in mid-pregnancy, and replacing the use of placebo with active treatment and avoiding the refusal to participate of the approximately one-third of eligible women observed in the research study, then screening 32 thousand asymptomatic pregnancies would identify 733 suitable women (2.3%) resulting in prevention of birth <33 weeks in 47 cases (14.7 per 10,000).

There is no doubt that any clinical strategy that would prevent the preterm birth of so many infants would be of considerable benefit to our patients and their families. The cost-effectiveness, however, would be dependent on the health-care environment and availability of appropriate resources and funds. In a decision analysis model comparing no routine cervical length screening with a single routine ultrasound trans-vaginal cervical length measurement at 18–24 weeks gestation, with the women with a short cervix then offered vaginal progesterone treatment, the policy appeared to be cost effective (49). In US dollars in the year 2010, for every 100,000 women screened, $12 million could be saved and 424 quality-adjusted life-years gained.

At this time, the American College of Obstetricians and Gynecologists has not recommended routine cervical length screening for all pregnancies, probably as a result of the large numbers requiring to be screened, and a perceived need for further studies to be conducted across a variety of health-care settings (47, 50). Practice guidelines are required in each clinical environment that enable the effectiveness of this protocol to be adopted within the resources and expertise that can be harnessed for the challenge. As our use of progesterone expands, we also need to remain mindful that we do not yet have data describing the complete safety of their use for later child and adult life.

Progesterone has also been evaluated as a potential treatment in other conditions that may lead to preterm birth. Studies conducted to-date have shown that progesterone treatment is not effective in preventing preterm birth in multiple pregnancies, preterm labor, or preterm pre-labor rupture of membranes (46, 47).

### Cervical cerclage

Cervical cerclage is the surgical placement of a suture or tape around the cervix in an attempt to prevent dilatation and subsequent preterm birth. The procedure was first described by Shirodkar in 1955 and involved dissection of the bladder superiorly to enable placement of the suture as close to the internal os as possible (51). A simplified procedure was described by McDonald 2 years later in which bladder dissection is not performed, minimizing the intervention but possibly leaving the suture lower in the cervix (52).

The decision to insert a cervical cerclage in mid-pregnancy is based on one of the three scenarios. First, a history of preterm births, classically recurrent and painless second trimester losses. Second, shortening of the cervix on ultrasound imaging. Third, short or dilated cervix on physical examination (13).

The mechanistic basis by which cerclage is effective is simplistically described as physical closure of the cervix, but the concept of cervical “incompetence” remains as much an enigma today as it was when described by Shirodkar in 1955 (51). As a result, the benefits of the procedure need to be carefully balanced against the potential risks and alternative therapies. The effects of cerclage on the cervico-vaginal microbiota may be clinically important, although has not yet been investigated.

#### Cerclage compared with no treatment

Meta-analysis of the RCTs that have compared cervical cerclage against no treatment has shown a significant reduction in preterm births of 20% (average RR 0.80, 95% CI 0.69–0.95) and with a reduction in perinatal deaths although this difference did not quite reach statistical significance (RR 0.78; 95% CI 0.61–1.00) (13). However, cerclage was associated with higher rates of fever, vaginal discharge, and vaginal bleeding, together with a significant increase in delivery by Cesarean section (RR 1.19, 95% CI 1.01–1.40).

#### Cerclage compared with progesterone

Only one trial has attempted to directly compare ultrasound-indicated cervical cerclage with a progesterone (53). The progesterone was 17P given intramuscularly. The trial was halted prematurely, and the sample size was too small to make meaningful conclusions (13).

No trial has compared vaginal progesterone versus cerclage for ultrasound-detected cervical shortening in mid-pregnancy (13). An indirect comparison using adjusted indirect meta-analysis of trials was reported by Conde-Agudelo et al. (54). The analysis included trials of singleton pregnancies with a history of previous preterm birth and in which ultrasound imaging had demonstrated a short cervix in mid-pregnancy. Both vaginal progesterone and cerclage were found to be effective in preventing preterm birth and improving perinatal outcomes. Neither treatment, however, was superior to the other.

At this time, the evidence guiding clinical practice in making a decision to insert a cervical cerclage versus administration of progesterone is incomplete. Decisions need to be based on informed consent and include patient and clinician preference, as well as the local availability of surgical resources and expertise.

#### Cerclage compared with pessary

Cervical pessaries have been proposed as an alternative method of preventing preterm birth (55). A range of designs has been proposed and some success has been described. At this time, however, the role of pessaries and the most effective clinical protocols for their use remain under investigation.

### Prevent cigarette smoking

Tobacco smoking in pregnancy causes preterm birth in addition to a dose-dependent reduction in birthweight (14, 56, 57). The exact mechanism by which preterm birth is triggered is uncertain and probably relates to the vasoconstrictive effects of nicotine, the increase in circulating levels of carbon monoxide, or other as yet unknown effects from the 4000 chemically active components in tobacco smoke. The woman herself does not need to be the smoker for there to be an increased risk of preterm birth. Second-hand smoke is associated with an increased risk of early birth, as well as stillbirth, low birthweight, and respiratory disorders in childhood (58).

The nicotine in cigarette smoke is addictive and produces the positive feelings inherent in addictive behaviors. Strategies aiming to prevent smoking in pregnancy, however, are complicated by the many factors that contribute to the decision-making processes in women who elect to smoke while pregnant. In high-income countries, rates of smoking in pregnancy have declined in recent years but the reduction has not been in all groups (59, 60). Smoking in pregnancy in high-income countries is now a marker of social disadvantage and remains common in many indigenous groups (61). There are also cultural factors that contribute, resulting in complex interplays between socio-economic disadvantage, social isolation, cultural background, migration, and mental health (62–64). Hence, anti-smoking campaigns that are effective in one demographic group may alienate others and be either ineffective or even be at-risk of producing an opposite effect.

Nevertheless, the risk of preterm birth attributable to smoking has been estimated as more than 25% and reducing smoking rates in pregnant women must be of highest priority (65). Psychosocial interventions appear to be moderately effective. Pooled data from 14 studies describing a variety of psychosocial interventions have shown a significant reduction in smoking rates (RR 0.82, 95% CI 0.70–0.96) (64). The number need to treat to prevent one case of preterm birth was 71.

Pharmacological interventions may be of less value. Nicotine replacement therapy is the only pharmacotherapy for smoking cessation in pregnancy that has been adequately tested. A review of the six published trials was unable to confirm benefit (66). Further studies involving different demographic groups and alternative agents are required.

In contrast, smoke-free legislation appears to be of great benefit (15). A review of 11 studies involving local or national bans and including more than 2.5 million births showed that smoke-free legislation was associated with a significant 10% reduction in preterm births (95% CI −18.8 to −2.0). The data in this analysis were observational rather than randomized, but the likelihood of causality was increased by the dose-dependent nature of the effect with comprehensive smoking laws appearing to produce the greatest benefit. It is likely that the major action on pregnancy outcomes from the legislative changes resulted from reductions in second-hand smoke effects. It is of interest that the reduction in preterm birth rates was not associated with a similar effect on rates of low birthweight. Maternal smoking in pregnancy is known to produce a dose-dependent reduction in birthweight, and it is possible that second-hand smoke exposure may act to trigger preterm birth acting through a more rapid and different pathway to chronic exposure to cigarette smoke where there is a most definite and consistent effect on fetal growth.

### Judicious use of fertility treatments

The advent of fertility assistance has contributed to a significant increase in the rate of preterm birth. Central to this contribution has been an increase in the incidence of multiple pregnancies. In USA, the incidence of multiple births has doubled from 1.8% of all births in 1972 to 3.5% in 2011 (67), and this rise can be attributed to an increase in the use of medically assisted reproduction. It has been estimated that in the US in 2011, 36% of twin births and 77% of triplet and higher order multiple births were due to medically assisted conception (67).

Data on *in vitro* fertilization (IVF) cycles in many countries are relatively easy to analyze; however, the data capture on less invasive treatments, such as ovulation induction and intra-uterine insemination cycles (often combined with ovarian stimulation) are less readily available. These treatments often involve ovarian stimulation and clinicians and patients may elect to proceed to fertility treatment when several follicles are potentially available for ovulation. In such circumstances, multiple pregnancies may result. In 2000, the incidence of twin gestations resulting from non-IVF fertility treatment was estimated to be 20% (68).

The risk of preterm birth that may result from fertility treatment can best be addressed by education of the attending health-care practitioners. The rate of multiple gestations resulting from IVF treatment can be reduced to relatively low levels. In Australia and New Zealand, single embryo transfer has been embraced widely resulting in a multiple pregnancy rate following IVF of only 6.9% (69). Transferring a single embryo minimizes the risk of multiple pregnancy but requires an environment of high competence and patient education as the overall pregnancy rate is less (16). In recent years, the percentage of embryo transfers that were single in Australia and New Zealand was 69%, compared to 40% in the UK (http://www.HFEA.gov.uk). Meanwhile, in USA, the percentage of single embryo transfers was 21%, resulting in IVF being responsible for one in five multiple births in that country in 2011 (67).

Currently, one in 25 children born in Australia has resulted from IVF procedures (70). In Denmark, the percentage is almost 5% (70). As the use of IVF technology spreads progressively across the world, measures are required to ensure that responsible ovulation induction treatment and a single embryo transfer approach in IVF treatment are embraced to minimize the risk of multiple pregnancies and risk of preterm birth.

Multiple pregnancies are not the only pathway by which fertility treatment can lead to preterm birth. It is well established that there are other obstetric and perinatal complications that may befall a mother and her infant as a result of IVF treatment (71, 72). At first, it was thought that additional perinatal risks resulted purely from complications of multiple pregnancies but data from several countries in which single embryo transfer is common have shown additional risks even in the presence of a single fetus. The causes of these additional risks are unclear. One contributing factor may be the underlying cause of the subfertility. Evidence for an effect of subfertility itself has come from observations that women with a history of subfertility who conceive spontaneously have a significantly worse perinatal prognosis than those with normal fertility (73–75), and women who require intra-uterine insemination have a significantly worse perinatal outcome than women who spontaneously conceive (74, 76). Ironically, some of the worst perinatal outcomes exist for women who conceive a singleton pregnancy as a result of IVF treatment, with an approximate doubling of the risk of stillbirth, growth restriction, preterm delivery, and neonatal nursery admission for their baby (71, 72, 77, 78). Hence, a woman with subfertility has an increased perinatal risk due to her subfertility. Further, the perinatal mortality of a single fetus conceived after a double embryo transfer procedure is significantly greater than a singleton conceived from a single embryo transfer (79).

Despite the trend toward single embryo transfer, the incidence of monozygotic twinning is believed to remain increased by IVF treatment by an additional 1–5% (80). The risk of monozygotic twinning is particularly increased by the procedures of assisted hatching (81) and blastocyst transfer in comparison to early embryo transfer (82). As a result, these procedures increase the risk of preterm birth.

Finally, children born as a result of assisted reproductive technology have an excess risk of birth defects when compared to spontaneously conceived children, further increasing the chance of obstetric intervention and preterm birth (83).

### Dedicated preterm birth prevention clinics

In recent years, many health regions and hospitals have developed dedicated preterm birth prevention clinics. These clinics and their associated services have employed a wide variety of criteria outlining who should attend and the protocols for management. The first large-scale attempt to determine the effectiveness of such a program was the West Los Angeles Preterm Birth Prevention Project in which eight prenatal county clinics in California were allocated at random to be experimental or control clinics (84). The intervention was based on providing additional education to the women and offering more clinic attendances. In the experimental group, there was a 19% reduction (9.1–7.4%) in the preterm birth rate when compared with that of the control clinics. This difference in rates was statistically significant when the number of patient risk factors was taken into account. In pregnancies of black women, the preterm birth rate was 15% in the experimental clinics and 22% in the control clinics. Secondary interventions of bed rest, social work assistance, and oral synthetic progesterone medication were of no additional benefit.

More recently, most dedicated preterm birth prevention clinics have focused on newer diagnostics and therapeutic interventions including assessment of vaginal microbiology, fibronectin testing, ultrasound detection of shortened cervix, antibiotic use, progesterone therapy, cervical cerclage, and Arabin cervical pessaries. A survey of 23 dedicated preterm birth prevention clinics in UK in 2012/13 revealed considerable heterogeneity in protocols and practices suggesting a need for effective networking and coordination of such services (85). Also, there was considerable variation in the criteria for referral and attendance, reflecting the fact that risk scoring systems have generally been unhelpful in predicting preterm birth (86). Most clinics attempt to target women with a history of preterm birth or recurrent mid-pregnancy loss, previous preterm pre-labor rupture of membranes, or previous loop excision or cone biopsy of the cervix (85).

Using a retrospective cohort design, investigators from Utah, USA reported a significant reduction in recurrent preterm birth (48.6 versus 63.4%) in women who attended a dedicated clinic and with lower rates of composite major neonatal morbidity (5.7 versus 16.3%) (87). The intervention was consultative and consisted of three standardized clinic attendances with routine prescription of 17-alpha hydroxyprogesterone caproate, as well as sonographic measurement of cervical length. Using a similar study design, investigators from Ohio, USA reported on the outcomes from their preterm birth prevention clinic after adoption of an accelerated appointment process and prophylactic treatment with progesterone (88). After adjustment for major confounders, these changes to their practice resulted in a significant 25% decrease in spontaneous preterm birth.

Despite the relatively large number of preterm birth prevention clinics now operating in various parts of the world, a review of their effectiveness could identify only three trials that qualified for inclusion in the analysis, and with only one study providing outcome data on most end-points (17). When data from the three trials were pooled, there were fewer preterm births in the treatment group compared with controls, but the difference between groups was not statistically significant (RR 0.87, 95% CI 0.69–1.08). The authors concluded that adequate randomized controlled trials of preterm birth prevention clinics may never be performed as such clinics have become an accepted part of antenatal care in many countries.

In addition to the enhanced provision of expert care and application of effective interventions for at-risk women, an important function of dedicated preterm birth prevention clinics may also be to alleviate maternal anxiety. Stress has been thought for many years to be a possible cause of some cases of early birth, and research is needed on the potential benefits or otherwise of attendance at such clinics (89).

## Strategies with Promise but Requiring More Research

### Treatment of intra-uterine infection

Intra-uterine infection and inflammation play a well-recognized role in the etiology of spontaneous preterm labor, particularly in deliveries less than 32 weeks gestation (90) or those complicated by preterm pre-labor rupture of membranes (91). The primary reservoir for such infection is the vagina. Vaginal microorganisms are hypothesized to breach the cervical barrier, colonize the fetal membranes, and eventually the amniotic cavity (91, 92). The vigorous inflammatory response ultimately leads to preterm birth.

The microorganisms most commonly isolated from the amniotic fluid are very small bacteria of the Class Mollicutes, namely *Ureaplasma* and *Mycoplasma* species (93). Numerous other bacteria have also been identified in infected amniotic fluid samples including *Streptococcus*, *Fusobacterium*, and *Enterobateriaceae*. The frequent presence of these organisms does not necessarily denote causation, but there is evidence from several sources to support a role for some of these organisms in the causal pathway to preterm labor. In experimental sheep, intra-amniotic injection of *Ureaplasma* spp. elicits a robust intra-uterine inflammatory response and enhanced lung maturation (94–96). Intra-amniotic injection with *Ureaplasma* spp. in chronically catheterized Rhesus macaques drives intra-uterine cytokine and prostaglandin production, chorioamnionitis and preterm labor, replicating the disease pathogenesis and ontogeny observed in human pregnancy (97).

The relationships between vaginal microbiota and ascending infection resulting in preterm birth remain uncertain. For several decades, investigators have explored a role for bacterial vaginosis. Bacterial vaginosis is a common genital condition among women of reproductive age characterized by a disturbance in normal vaginal microbiota with a loss of H_2_O_2_-producing *Lactobacillus* spp., an increase in vaginal pH, and an increase in Gram-variable cocco-bacilli, anaerobic organisms, and genital mycoplasmas (98). There are well-established associations between bacterial vaginosis and preterm birth (99), but the extent of a causative role is not certain. What is known with certainty, however, is that bacterial vaginosis varies dramatically with race and that studies need to be specific for different population groups (98, 100).

Antibiotic treatment of bacterial vaginosis is generally ineffective in preventing preterm birth (101–103). It should be noted, however, that antibiotics commonly used to treat bacterial vaginosis are ineffective against *Ureaplasma* and *Mycoplasma* spp., which are the organisms most frequently associated with preterm birth. These organisms are best treated with macrolide antibiotics, the most frequently used of which are erythromycin and azithromycin. In addition, the transplacental passage of these drugs is poor and unlikely to reach levels sufficient to eradicate infection (104–107). Newer macrolide antibiotics, such as solithromycin, with greater efficacy and better transplacental passage, offer promise and may prove in time to be more effective (108, 109).

There is some evidence, however, that treatment earlier in pregnancy may be more effective in preventing preterm birth. Lamont and colleagues have shown that administration of clindamycin to women with abnormal vaginal flora before 22 weeks gestation may reduce the rate of subsequent preterm birth (110). This possibility is the topic of a separate review in this series where it will be discussed at length.

In summary, while a role for vaginal infection in the causal pathway to many cases of early preterm birth seems clear, at this time translation of that knowledge into an effective treatment strategy has yet to be widely adopted. The field is the subject of active investigation and progress can be anticipated in the near future.

### Nutritional interventions

There has been considerable research on the interactions between nutrition and risk of preterm birth, but the many environments and demographic groups included in these studies complicates interpretation. A low pre-pregnancy body mass index (BMI) has been associated with an increased risk of preterm birth, while obesity has been shown to be protective (111). Obesity, however, predisposes pregnant women to diabetes and pre-eclampsia often leading to iatrogenic early birth.

It has been known for many years that there are associations between preterm birth and low serum levels of many micronutrients. Proving a causative role and using supplementation to reduce preterm birth rates has, however, so far remained elusive.

In a large cohort study, a strong association was observed between use of pre-conception folate supplementation for one year or more and reduction in risk of preterm birth before 32 weeks gestation, but not at later gestational ages (112). However, the randomized controlled trials designed to investigate the effects of peri-conception folate supplementation on rates of neural tube defects did not reveal any reductions in rates of miscarriage or low birthweight (113). It is entirely possible that observational studies of folate use may describe a population of women at lower risk of preterm birth for other reasons. Further research is required before peri-conception folate supplementation can be considered to be an effective strategy to prevent preterm birth.

Research is underway investigating the possibility that maternal intake of omega-3 long-chain polyunsaturated fatty acids may prevent preterm birth and improve birth weight. The impetus for this hypothesis was the observation that women living in the Faroe Islands who have a high consumption of fish oil also have pregnancies of longer gestational ages and infants of high birth weight (114). A recent randomized controlled trial using the *n* – 3 (omega-3) long-chain polyunsaturated fatty acid docosahexaenoic acid (DHA) in the last half of pregnancy resulted in fewer preterm births before 34 weeks gestation, longer gestations and shorter hospital stay for preterm infants (115). Such results are encouraging but further research is required before supplementing the maternal diet with omega-3 fatty acids to prevent preterm birth can be recommended (116).

### Amelioration of maternal stress

It has been shown that women with high levels of psychological or social stress are at increased risk of preterm birth (117–119). Randomized controlled trials of interventions aiming to relieve stress or provide comforting reassurance have not been successful in preventing early birth suggesting that multiple other confounding factors are contributing to the relationship between stress and preterm birth (120).

### Treatment of periodontal disease

It has been known for many years that periodontal disease is associated with preterm birth (121). Inflamed and infected periodontal tissues could stimulate preterm labor either by translocation of periodontopathic organisms, or by stimulation and release of inflammatory mediators and prostaglandins into the maternal circulation (122). Disappointingly, randomized controlled trials of treating periodontal disease during pregnancy have failed to lower the rate of preterm birth (123–125). It seems likely that the alterations in maternal immune responses that cause periodontal disease also predispose women to preterm birth, but that treating periodontal disease during pregnancy will neither cause nor prevent this major complication of pregnancy. Nevertheless, further research is required to investigate if there is any benefit in reducing rates of preterm birth by treatment of periodontal disease before conception as the randomized controlled trials conducted so far have initiated treatment in mid-pregnancy.

### Prevention of surgical treatment for cervical intra-epithelial neoplasia

It is well established that surgical treatments of cervical intra-epithelial neoplasia (CIN) predispose women to preterm birth in subsequent pregnancies, including early preterm birth (126). Such treatments aim to prevent cancer of the cervix and the possible risks for future pregnancies have always been judged against the need to prevent life-threatening cancer. It has now been shown that the vast majority of cases of CIN are caused by human papillomavirus (HPV) infection (127). The discovery and introduction of a vaccine to prevent HPV infection can now be expected to dramatically reduce the prevalence of pre-invasive abnormalities of the cervix and hence decrease the need for surgical treatments that may predispose women to subsequent preterm births (128). In addition to saving lives of young women who are vaccinated, it is likely that time will show that discovery of the vaccine to prevent HPV infection will have serendipitously improved outcomes for the next generation by also preventing early birth. Population-based vaccination of young women to prevent HPV vaccination needs to be given high priority.

## Conclusion

In recent decades, advances in newborn care have resulted in improved outcomes for large numbers of children who have been born too early but this progress has not been matched by similar advances in our ability to prevent preterm birth. Times have changed. We now have increasing evidence that a variety of interventions have potential to significantly and safely prevent a meaningful proportion of preterm births. Translation of recent discoveries into clinical practice will have different requirements for high and low-resource settings, and for different population groups. For each setting, the best chance of success will come from an integrated implementation strategy that harnesses both the health-care personnel and the pregnant women for whom they provide care. The interventions are in many cases multi-disciplinary and require the participation of personnel from multiple fields including those who make local and national policies. This process begins with awareness across the medical and general communities that preterm birth is one of modern health care’s greatest challenges, but that prevention in many cases is now possible.

## Conflict of Interest Statement

The authors declare that the research was conducted in the absence of any commercial or financial relationships that could be construed as a potential conflict of interest.
